# Effect of Diet on the Growth Performance, Feed Conversion, and Nutrient Content of the House Cricket

**DOI:** 10.1093/jisesa/ieaa014

**Published:** 2020-03-27

**Authors:** M Bawa, S Songsermpong, C Kaewtapee, W Chanput

**Affiliations:** 1 Faculty of Agro-Industry, Department of Food Science and Technology, Kasetsart University, Chatuchak, Bangkok, Thailand; 2 Faculty of Agriculture, Department of Animal Science, Kasetsart University, Chatuchak, Bangkok, Thailand

**Keywords:** crickets rearing, crickets feed, nutritional value, pumpkin

## Abstract

The house crickets, *Acheta domesticus*, are sustainable and nutritious future sources of food, due to their nutritional benefits, particular high protein content and potential in solving global malnutrition. Different diets, particularly protein content, can influence the growth and nutritional value of crickets. The aim of this present study was to evaluate the effects of commercial diets and other formulated diets on the nutritional composition and growth parameters of the house crickets, being a major challenge to cricket’s farmers in Thailand. Feed conversion ratio were 1.50, 1.50, and 1.51 for fed crickets on a blend of 22% protein and dry pulp pumpkin powder, fed 22% protein plus fresh pumpkin pulp, and fed 22% protein alone, indicated that these groups are high feed convertors and represented the quality of these diets compared to 1.73 and 1.81 for fed crickets on a blend of 22% and 16% protein, and those fed on 16% protein alone. Fed crickets on 22% protein had the highest amount of protein (76%), the lowest (48%) in those fed on 22% protein and fresh pumpkin pulp inclusion. The group on 22% protein diet also had the highest amount of phosphorus, potassium, calcium, and sodium. Fed 22% protein and either dry pulp pumpkin powder or fresh pumpkin pulp condition have shown improvement in vitamin B content. Crickets can effectively be produced on 22% protein diet to improve yield output and several minerals such as phosphorus, potassium, calcium, and sodium. In contrast, the supplementation of 22% protein diet with pumpkin (*Cucurbita maxima*) will improve vitamin B content.

The United Nations Food and Agricultural Organization (FAO) estimated that global population growth may exceed food sources available for 9 billion people by 2050. This may double the impact of global malnutrition and diet-related diseases. Currently, 155 million under-five children are stunted, 52 million are wasted, and 27.6 million of malnourished children living in southern Asia (FAO/WHO 2017). Interventions tacking global malnutrition and promoting improved nutrition of population can only be achieved, with sustainable food sources. The house crickets are sustainable and nutritious source of food (DeFoliart 1999, Collavo et al. 2005, Oonincx et al. 2015), whereas conventional animals and crop production have environmental and sustainability problems (FAO 2013). Crop production accounts for 70% of fresh water, livestock production also accounts for 30% of crop yield and 8% of water usage (Premalatha et al. 2011). Further, one of the benefits in rearing crickets has been their less environmental impact, with 0.05 carbon dioxide (g/kg body mass/day) and 5.4 ammonia gas (mg/kg body mass/day) production compared to 2–28 carbon dioxide (g/kg body mass/day) and 5–57 ammonia gas (mg/kg body mass/day) contribution from pigs and 5.98 carbon dioxide (g/kg body mass/day) and 14–170 ammonia gas (mg/kg body mass/day) emission from cattle (Groot Koerkamp et al. 1998, Harper et al. 2009, Oonincx et al. 2010). Entomophagy has been a major source of nutrition for over 2 billion people, with a global coverage of 80% (van Huis et al. 2013). While 2,000 different edible insects have been identified as human nutritious food (FAO 2013). In Thailand, *Acheta domesticus* is one of the edible insect species that can easily be found in Seven Eleven shops and traditional markets. They are the major farmed crickets in Thailand, used as a nutrient source for food, due to its preferred taste and texture (Hanboonsong et al. 2013). Recently, its significance as nutritious sources of feed for animals has also been highlighted by the fact that, it is listed among the species that can be included in aquafeeds in the EU (EU Regulation 2017/893). The potential for their use, as a dietary supplement, aimed at meeting the recommended dietary intake (RDI) of population has been established (FAO 2013). For example, a 100 g of crickets can provide 63 g protein, 447 kcal of energy, 0.25 g omega-3 fatty acids, and 5.0 mg iron compared to 25.6 g protein, 278 kcal, 0.009 g omega-3 fatty acids, and 2.4 mg iron in 100 g of beef, and 39 g protein, 190 kcal, 0.05 g omega-3 fatty acids, and 1.2 mg iron in 100 g of chicken (Finke 2002). In addition, evidence of the nutritional importance of crickets comes from the study of Rumpold and Schlüter (2013), who showed that iron and B12 status can be improved with crickets. However, cricket’s nutritional profile is closely related to the diets, due to its influence (McFarlane et al. 1959, Finke et al. 1989, Oonincx et al. 2019). Whereas the diet of the house crickets is essential for its growth and development (Orinda et al. 2017, Sorjonen et al. 2019). Crickets survival rate, feed efficiency, and development time can also be influenced by different diets, especially protein, carbohydrate, and fat content of insect diets (Oonincx et al. 2015). Therefore, interventions to address global malnutrition, and food insecurity through the promotion of *A. domesticus*, will require mass rearing, formulation of suitable diets, and improved feeding techniques. However, literature on the diet of this species is old (McFarlane et al. 1959, Patton 1967, Nakagaki and Defoliart 1991), whereas relevant recent research articles are limited (Lundy and Parrella 2015). Therefore, the present study aims to fill this gap. Especially, since the diets tested include commercial diets used in mass rearing in Thailand. Additionally, research findings documented so far do not include the type of diets and feeding methods in Thailand. During field interviews with cricket’s farmers in Thailand, they reported that diet can affect the growth parameters and nutritional profile of the house crickets. Farmers also indicated that addition of fresh pumpkin pulp to cricket diets will improve nutrient contents. Therefore, cricket diet and formulation can affect food security and nutritional well-being of individuals and population. Scientific knowledge should be applied in mass rearing to effectively improve nutrient contents and yield output.

This highlighted the need for a feed experiment to study the effects of these diets on several growth parameters, mean body weight, length, width, feed efficiency, and survival rate and assess the influence of the diet on nutritional composition of the house crickets. We were also inclined to provide additional evidence to support mass rearing of *A. domesticus*. Due to sparseness of data on pumpkin pulp inclusion in commercial diets and its effects on growth parameters and nutritional composition of *A. domesticus*. Cricket’s farmers’ hypotheses that the supplementation of cricket’s diets with pumpkin pulp will improve nutrients contents and growth parameters of *A. domesticus* were also evaluated.

## Materials and Methods

### Feed Formulation and Comparison

To evaluate the effects of commercial diets on the nutritional composition and growth parameters of the house crickets. Two major diets, Pure pride crickets feed was purchased (Pure pride cricket feed 7001, Thai feed mill, Bangkok, Thailand), and Betagro chicken feed was purchased (Betagro 215, Betagro, Bangkok, Thailand). Pure pride crickets feed (PPF or 22% protein feed) consisted of fish meal, soybean meal, ground *Acacia* leaves, corn meal, rice bran, vegetable oil, dicalcium phosphate, salt, vitamins, minerals, and amino acids. Betagro chicken feed 215 (BF or 16% protein feed) consisted of fish meal, meat, ground bone, soybean meal and/or sun flower seed meal, corn meal and/or millet and/or broken rice and/or cassava, rice bran and/or defatted rice bran, molasses, vegetable oil, calcium carbonate and/or dicalcium phosphate, salt, vitamins, minerals, and amino acids. The fresh pumpkin (*Cucurbita maxima*) was purchased from a local market (Tesco Lotus, Co. Ltd., Bangkok, Thailand).

The diet assessment consisted of two major commercial diets used in mass rearing: the first commercial diet was 250 g of Pure pride crickets feed (PPF or a 22% protein diet); the second commercial diet was 250 g of Betagro chicken feed (BF or 16% protein diet). Other diets formulated included 250 g PPF (22% protein diet) supplemented with 100 g fresh pumpkin pulp per day (this diet was classified as PPFP or 18% protein diet); second diet formulated consisted of a mixture of 250 g PPF (22% protein diet) and 100 g dry pulp pumpkin powder per day that were thoroughly mixed together (this diet was classified as PPDP or 20% protein diet); the third diet consisted of a mixture of 50% Pure pride crickets feed (22% protein diet) and 50% Betagro chicken feed (16% protein diet) thoroughly mixed together (this diet was classified as PB or 19% protein diet). A 100 g fresh pumpkin pulp or 100 g dry pulp pumpkin powder was added as an additional supplement (additional benefit), without replacing the commercial diets to ensure that additional benefits of the supplementation are captured. The 100 g fresh pumpkin pulp or 100 g dry pulp pumpkin powder was added as an additional supplement (additional benefit), without replacing the commercial diets to ensure that additional benefits of the supplementation are captured. While the supplementation resulted in 350 g daily diet provision for fed crickets on PPFP and PPDP compared to 250 g provision for the other treatments. The data were comparable since these dietary assessments were aimed at providing further scientific evidence to support the inclusion of fresh pumpkin pulp or dry pulp pumpkin powder in commercial diets (exposure to pumpkin without accounting for protein loss by replacing the commercial diets with pumpkin), being a common practice in Thailand. The pumpkin is normally used due to its availability and prices are affordable. We also ensure that all groups had enough diets (even groups who were given 250 g diets each day without 100 g fresh pumpkin pulp or 100 g dry pulp pumpkin powder had leftovers) so that any treatment effects cannot be associated to a limiting diet. For dried pumpkin powder, 200 g of the fresh pumpkin pulp was placed in a ceramic bowl and dried at 600 W for 8 min at 2 min regular intervals, using a domestic microwave oven (Sharp, R-380I, Thailand). The dry pumpkin pulp was then milled into powder, using a heavy-duty blender (Otto, BE-127A, Thailand).

### Crickets Rearing

The house crickets, *A. domesticus*, were reared on the fifth floor at the Department of Food Science and Technology, Faculty of Agro Industry, Kasetsart University, Thailand. All feeding experiments were conducted, using newly hatched nymphs kept in 50 cm × 50 cm × 60 cm wooden frame which have emerged from *A. domesticus* eggs, obtained from a commercial cricket’s breeder in Thailand (Nonthaburi cricket’s farm, Nonthaburi, Thailand). The nymphs were randomly assigned to five feed treatments on the first day of emergence. The breeding house was purchased (Nonthaburi cricket’s farm), and consisted of a wooden frame; the tops of the breeding houses were fixed with net to allow ventilation; the foots of each breeding house were placed in vegetable oil traps to prevent ants and other predators from gaining access to the breeding houses. Fifteen egg board cartons were placed in each breeding house to provide a place for the crickets to hide. The crickets were fed every day through a 25 cm × 25 cm feeding trough made with wooden frame. Each breeding house was equipped with one feeding trough and one water trough. Three hundred milliliters of water was provided every day per diet treatment through 50 cm × 5 cm water trough made with PVC pipe with 10 holes on its surface filled with thread to allow the crickets to sip water through the thread. Each breeding house was equipped with 1,000 nymphs from two containers placed in each breeding house. To determine the effects of the diet treatments on crickets mean body size: 50 crickets were randomly sampled per diet treatment, the individual cricket was weighed with analytical balance (Metler Toledo, XP205, Switzerland). House cricket’s body size measurements were taken every day until 48th day, when crickets have fully developed wings. The weight, length, and width measurements were taken from 50% male and 50% female on 28th day, when the female ovipositor was visible for all diet treatments but was not fully developed. The length (mm) measurements were taken every day with a Vernier calliper (Womdee 6 inch/150mm Digital Caliper, Womdee, China) from the tip of the head to the tip of the abdomen. Width (mm) measurements were taken from the tip of the midpoint of the thorax to the tip of the corresponding midpoint of the thorax in horizontal plane. Weight, length, and width measurements were taken separately from the same individual sample. The cricket’s farm was equipped with a digital hygrometer with thermometer (VIP Digital LCD Thermometer Hygrometer Temperature, HTC-1, China). Temperature and humidity were recorded at the time, when body size measurements were taken around 8:00 a.m. The mean temperature and relative humidity were 28.92 ± 1.01°C and 67.50 ± 2.89%, respectively.

To evaluate the effect of the diet treatments on feed conversion ratio (FCR): 250 nymphs were divided into five groups (50 nymphs per group) and were kept in a separate breeding house. They were randomly subjected to the five diet treatments under comparison in a condition that was similar to the 1,000 nymphs. Each group was provided with enough diet (100 g of each diet treatment) and enough water (100 ml of water every day). All the groups (50 crickets per group) were weighed separately every day as reported by Orinda et al. (2017). The amount of feed consumed per group was also weighed. The FCR was subsequently obtained by dividing the total diet consumed/total weight gain. The number of crickets was maintained with cricket’s sample kept in the same type of breeding house conditions as in 1,000 nymphs. Because it was possible to calculate the survival rate (%) at the end of the experiment. The 1,000 newly hatched nymphs on each of the diet treatment were observed daily to confirm and quantify the number of dead crickets. The survival rate (%) was then calculated by dividing number of crickets alive on 48th day/total number of crickets (1,000 crickets) × 100. The cost of feeding crickets per diet treatment was also evaluated: Cost of feeding per kg live weight gain = diet cost per kg × FCR (for example, Cost of 30 kg of Betagro feed is 350 Thai Baht, the cost of 1 kg of Betagro feed is 11.7 Thai Baht × the FCR. The mean cost of feeding per kg live weight gain for each diet treatment was calculated by multiplying the FCR obtained from the first, second, and third replications × diet cost per kg. The average cost of feeding per kg live weight gain was then calculated). Feed intake (kg) or quantity of feed consumed (kg) was obtained by recording the amount of feed consumed (g) each day. For every diet treatment three replicates data were obtained from the crickets mean body size measurements, FCR, amount of feed consumed, survival rate, cost of feeding, and the nutritional analysis. The first feeding experiment was conducted from April to June 2018, the second feeding experiment from July to September 2018, and the last feeding experiment from January 2019 to March 2019.

### Nutritional Analysis

#### Sample preparation

All crickets were harvested on 49th day; prior to harvesting crickets, they were starved for 8 h to empty their gut but were denied access to available water for 4 h. A 30-min freezing step was applied for crickets to go into a state of hibernation before freezing them with liquid nitrogen (1:1 w/w). The crickets were kept in polyethylene bags well tight to prevent exposure to moisture and air. Each bag was stocked with 250 g male crickets and 250 g female crickets and stored at −18°C. The frozen crickets, experimental diets, fresh pumpkin pulp, and dry pulp pumpkin powder were sent to a certified lab, Thai central lab, in Bangkok for nutritional composition analysis. All nutritional analyses were conducted in triplicates.

#### Proximate analyses

The proximate composition of crickets on all the different diet treatments was analyzed according to the Association of Official Analytical Chemist (AOAC 2016) standard procedures and methods. Crude protein content by Kjeldahl method with N × 6.25 (method 992.15), crude fiber (method 985.29), crude fat (method 922.06), moisture content (method 925.10). Carbohydrate content was estimated by the method of difference, following the procedures of Food and Agriculture Organization (FAO 2003). Crickets ash content was determined by AOAC (method 923.03).

#### Analyses of mineral content

Crickets calcium, zinc, potassium, phosphorus, and iron contents were determined according to the procedures by Poitevin (2012).

#### Analyses of vitamin content

Crickets vitamin A content was quantified following the method by DeVries et al. (2012). Vitamin B1, B2, B3, and B6 were estimated following the methods and procedures by Salvati et al. (2016). B12 was quantified according to the methods and procedures by Giménez and Martin (2018).

### Statistical Analysis

One-way ANOVA was conducted to assess the influence of the diet treatments on cricket’s body weight, length, survival rate, and feed efficiency. Post hoc multiple comparison, Tukey’s method, was conducted to evaluate differences among means for data that were normally distributed and had equal variances. However, Games Howell method was utilized to compare the mean crude fat content in house crickets due to unequal variances. Pearson’s correlation was used to examine the correlation between nutrient contents of the different diets and nutrient contents in the house crickets. Data were analyzed with Microsoft SPSS software version 17 (IBM, New York, USA). A *P-*value < 0.05 was considered statistically significant.

## Results

### Composition of Experimental Diet

The diet treatments differ significantly in crude protein, crude fat, crude fiber, crude ash, and carbohydrate contents (Table 1): PPF (22% crude protein) had the highest amount of crude protein (*F*_6,14_ = 7679216.54, *P* < 0.001), the lowest in FP (6% crude protein); BF (5% crude fat) had the highest amount of crude fat (*F*_6,14_ = 57607.12, *P* < 0.001), the lowest in PPF (2% crude fat) and FP (2%); DP (7% crude fiber) had the highest amount of crude fiber (*F*_6,14_ = 1569.00, *P* < 0.001), the lowest in BF (5% crude fiber); BF (8% crude ash) had the highest amount of crude ash (*F*_6,14_ = 762.89, *P* < 0.001), the lowest in FP (6%) and DP (6%); FP (79 % carbohydrate) had the highest amount of carbohydrate (*F*_6,14_ = 608642.86, *P* < 0.001), the lowest in PPF (65% carbohydrate) and PB (65% carbohydrate).

**Table 1.** Chemical composition of experimental diet (% dry matter basis)

Feed treatment	DM	Crude protein	Crude fat	Crude fiber	Crude ash	Carbohydrate
PPF	91.3 ± 0.01^a^	21.9 ± 0.01^a^	1.4 ± 0.01^g^	5.9 ± 0.03^e^	5.7 ± 0.01^c^	65.0 ± 0.02^f^
PB	98.9 ± 0.02^b^	18.9 ± 0.02^c^	3.4 ± 0.02^c^	5.6 ± 0.02^f^	6.7 ± 0.13^b^	65.3 ± 0.01^g^
BF	88.7 ± 0.03^d^	15.8 ± 0.02^e^	5.2 ± 0.01^a^	5.3 ± 0.02^g^	7.9 ± 0.06^a^	65.6 ± 0.01^e^
FP	11.3 ± 0.01^g^	6.3 ± 0.01^g^	2.3 ± 0.01^d^	6.4 ± 0.03^b^	5.6 ± 0.02^c^	79.4 ± 0.01^a^
DP	83.5 ± 0.01^e^	13.6 ± 0.02^f^	4.1 ± 0.01^b^	6.7 ± 0.03^a^	5.6 ± 0.01^c^	70.1 ± 0.01^b^
PPFP	68.5 ± 0.01^f^	17.5 ± 0.01^d^	1.7 ± 0.01^f^	6.0 ± 0.01^d^	5.7 ± 0.01^c^	69.1 ± 0.02^c^
PPDP	89.1 ± 0.03^c^	19.6 ± 0.02^b^	2.2 ± 0.01^e^	6.1 ± 0.01^c^	5.7 ± 0.01^c^	66.5 ± 0.01^d^

^*a*^Values are mean ± SD of triplicate analysis.

^*b*^Dry matter content (DM).

^*c*^Fresh pumpkin pulp (FP).

^*d*^Dried pulp pumpkin powder (DP).

^*e*^PPF (Pure pride).

^*f*^PB (diet consisted of a mixture of 50% Pure pride and 50% Betagro).

^*g*^BF (Betagro).

^*h*^PPFP (diet consisted of Pure pride supplemented with 100 g fresh pumpkin pulp per day).

^*i*^PPDP (diet consisted of a mixture of Pure pride and 100 g dry pulp pumpkin powder per day).

^*j*^Mean values with different superscripts letters in a column are significant at the 0.05 level.

### Crickets Mean Body Size, Quantity of Feed Consumed (kg), FCR, Survival Rate (%), and Cost of Feeding per kg Live Weight Gain

**Fig. 1.**
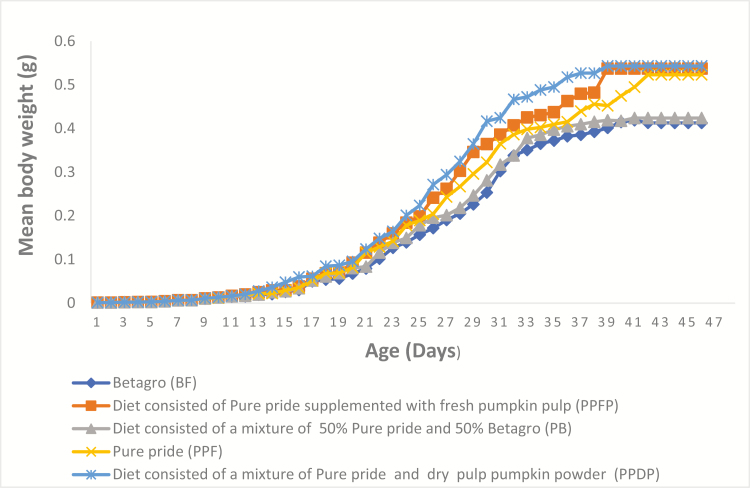
Mean body weight (g) of *Acheta domesticus* on two commercial diets, PPF (22% protein diet) and BF (16% protein diet) and other formulated diets, PB (19% protein diet), PPFP (18% protein diet), and PPDP (20% protein diet).

**Fig. 2.**
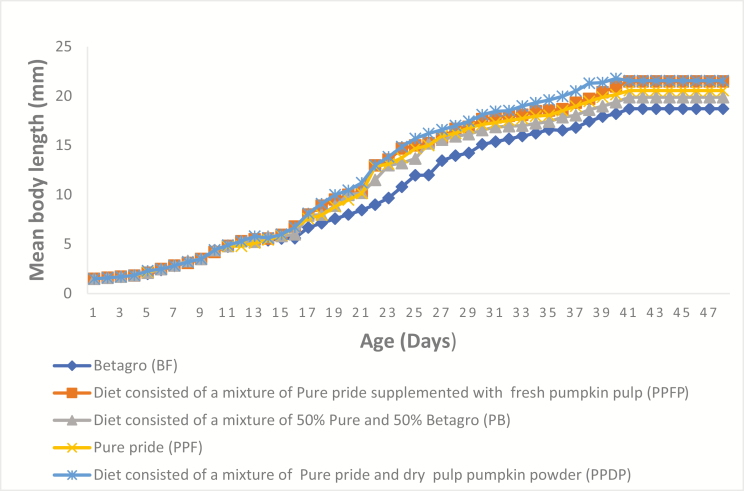
Mean body length (mm) of *Acheta domesticus* on two commercial diets, PPF (22% protein diet) and BF (16% protein diet) and other formulated diets, PB (19% protein diet), PPFP (18% protein diet), and PPDP (20% protein diet).

**Fig. 3.**
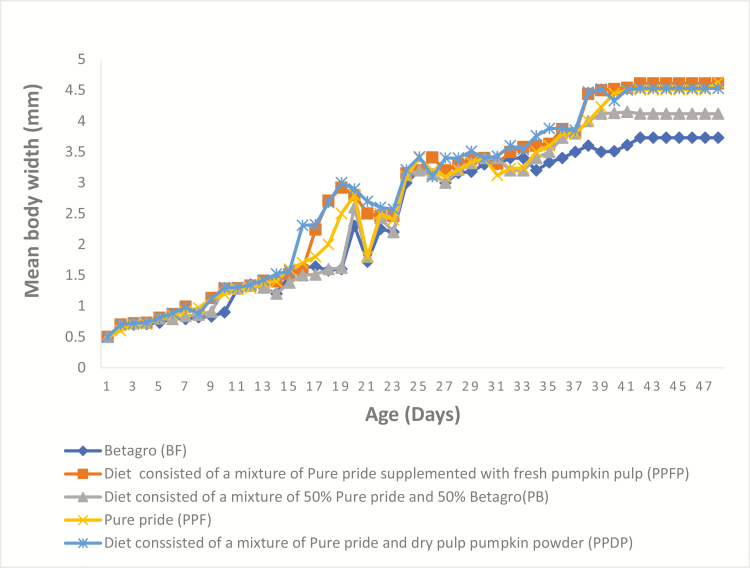
Mean body width (mm) of *Acheta domesticus* on two commercial diets, PPF (22% protein diet) and BF (16% protein diet) and other formulated diets, PB (19% protein diet), PPFP (18% protein diet), and PPDP (20% protein diet).

The mean weight of all newly emerged nymphs was 0.0006–0.0007 g from day 1 to 2; mean body length and width at the beginning of the feeding experiment were 1.5 ± 0.0 mm and 0.5 ± 0.0 mm, respectively (Table 2). The five curves in Figs. 1–3 show the patterns of growth of the crickets on the five experimental diets. From day 1 to 11 (about weeks), the five curves show similar trend, and after 11th day, crickets can be differentiated by diet treatments. Fed crickets on PPDP, PPFP, and PPF had better growth rates compared to crickets on the other treatments. However, there were a lot of variations in the growth curve obtained from crickets mean body width (Fig. 3). The different diet treatments resulted in significant higher mean body weight (*F*_4,10_ = 124.48, *P* < 0.001), mean body length (*F*_4,10_ = 25.69, *P* < 0.001) in fed crickets on PPF, the lowest in BF. Low FCR values (*F*_4,10_ = 301.34, *P* < 0.001) were obtained from the group on PPF, PPFP, and PPDP, the highest in fed crickets on BF (Table 2). However, no significant differences were observed for the number of crickets that survive on the 48th day (*F*_4,10_ = 2.19, *P* = 0.142). We found that high crude protein content in insect diets resulted in low FCR value (*r* = −0.618, *P* = 0.266).

**Table 2.** Cost of feeding per kg live weight gain, crickets mean body size, FCR and quantity of feed consumed of the house crickets on two commercial diets, PPF (22% protein diet) and BF (16% protein diet) and other formulated diets, PB (19% protein diet), PPFP (18% protein diet), and PPDP (20% protein diet)

Diet treatment	Mean body weight (g)	Mean body length (mm)	Quantity of feed consumed (kg)	FCR	Survival rate	Cost of feeding per kg live weight gain (Thai Baht)
PPF	0.523 ± 0.005^a^	20.56 ± 0.22^c^	4.52 ± 0.01^bc^	1.51 ± 0.01^c^	96 ± 2.1^a^	20.06 ± 0.15^d^
PB	0.422 ± 0.014^b^	19.89 ± 0.03^b^	4.55 ± 0.01^a^	1.73 ± 0.03^b^	95 ± 2.1^a^	21.47 ± 0.35^d^
BF	0.419 ± 0.011^b^	18.72 ± 0.37^d^	4.56 ± 0.01^a^	1.81 ± 0.02^a^	92 ± 3.8^a^	21.58 ± 0.32^c^
PPFP	0.537 ± 0.008^a^	21.48 ± 0.61^a^	4.54 ± 0.01^ac^	1.50 ± 0.01^c^	96 ± 1.5^a^	26.40 ± 1.04^b^
PPDP	0.543 ± 0.005^a^	21.55 ± 0.51^a^	4.50 ± 0.01^b^	1.50 ± 0.01^c^	97 ± 1.5^a^	28.94 ± 0.49^a^

^*a*^Values are mean ± SD of triplicate analysis.

^*b*^PPF (Pure pride or 22% protein diet).

^*c*^PB (diet consisted of a mixture of 50% Pure pride and 50% Betagro or 19% protein diet).

^*d*^BF (Betagro or 16% protein diet).

^*e*^PPFP (diet consisted of Pure pride supplemented with 100 g fresh pumpkin pulp per day or 18% protein diet).

^*f*^PPDP (diet consisted of a mixture of Pure pride and 100 g dry pulp pumpkin powder per day or 20% protein diet).

^*g*^Mean values with different superscripts letters in a column are significant at the 0.05 level.

### Proximate Composition

Proximate composition in crickets was significantly influenced by the diet treatments: Significantly higher quantity of crude protein (*F*_4,10_ = 855335.63, *P* = 0.001) was obtained from fed crickets on PPF, the lowest in crickets on PPFP. Significant differences were found in the dry matter (*F*_4,10_ = 116395.23, *P* < 0.001), crude fat (*F*_4,10_ = 735690.66, *P* < 0.001), crude fiber (*F*_4,10_ = 20741.42, *P* < 0.001), crude ash (*F*_4,5_ = 5031932.58, *P* < 0.001), and carbohydrate (*F*_4,10_ = 57706.66, *P* < 0.001) contents in crickets (Table 3). The Pearson’s correlation analysis revealed that high protein content in crickets resulted in low fat content in crickets (*r* = −0.987, *P* = 0.001).

**Table 3.** Crude Protein, crude fat, crude ash, crude fiber, carbohydrate (g/100 g dry matter basis), and dry mater (DM) content (% dry matter basis) of the house crickets on two commercial diets, PPF (22% protein diet) and BF (16% protein diet) and other formulated diets, PB (19% protein diet), PPFP (18% protein diet), and PPDP (20% protein diet)

Diet treatment	DM	Protein	Fat	Ash	Fiber	Carbohydrate
PPF	29.89 ± 0.03^c^	76.19 ± 0.01^a^	8.9 ± 0.06^e^	4.6 ± 0.02^a^	3.7 ± 0.02^e^	10.2 ± 0.07^b^
PB	29.54 ± 0.02^d^	71.02 ± 0.03^c^	19.3 ± 0.02^b^	4.4 ± 0.02^d^	6.4 ± 0.02^c^	5.2 ± 0.14^d^
BF	31.65 ± 0.03^b^	70.38 ± 0.02^d^	14.8 ± 0.01^c^	4.5 ± 0.02^b^	7.5 ± 0.03^a^	10.3 ± 0.08^a^
PPFP	40.55 ± 0.02^a^	48.06 ± 0.04^e^	43.9 ± 0.01^a^	2.9 ± 0.02^c^	4.5 ± 0.02^d^	5.1 ± 0.12^e^
PPDP	29.25 ± 0.01^e^	75.79 ± 0.11^b^	12.9 ± 0.02^d^	4.5 ± 0.01^b^	6.6 ± 0.02^b^	6.8 ± 0.16^c^

^*a*^Values are mean ± SD of triplicate analysis.

^*b*^Dry matter content (DM).

^*c*^PPF (Pure pride or 22% protein diet).

^*d*^PB (diet consisted of a mixture of 50% Pure pride and 50% Betagro or 19% protein diet).

^*e*^BF (Betagro or 16% protein diet).

^*f*^PPFP (diet consisted of a mixture of Pure pride supplemented with 100 g fresh pumpkin pulp per day or 18% protein diet).

^*g*^PPDP (diet consisted of a mixture of Pure pride and 100 g dry pumpkin pulp per day or 20% protein diet).

^*h*^Mean values with different superscripts letters in a column are significant at the 0.05 level.

### Vitamin Content of the House Crickets

Crickets vitamin B content was significantly influenced by the different diet treatments. Those fed on PB had the highest amount of B2 (*F*_4,10_ = 18719.54, *P* < 0.001), followed by fed crickets on PPFP, whereas those fed on BF and PPFP were the lowest. Those fed on PPDP had the highest amount of B12 (*F*_4,10_ = 530118, *P* < 0.001), the lowest in fed crickets on PPFP, whereas fed crickets on PPFP had the highest amount of B3 (*F*_4,10_ = 18483.44, *P* < 0.001), the lowest in BF (Table 4). It is worth noting that all the vitamin B groups analyzed in the present study were detected in fed crickets on PPFP. However, vitamin A was not detected in all treatments.

**Table 4.** Vitamin content (mg/100 g dry matter basis) of the house crickets on two commercial diets, PPF (22% protein diet) and BF (16% protein diet) and other formulated diets, PB (19% protein diet), PPFP (18% protein diet), and PPDP (20% protein diet)

Diet treatment	B1	B2	B3	B6	B12	Vitamin A
PPF	<0.020 ± 0.0^a^	–	1.33 ± 0.18^c^	–	0.31 ± 0.14^c^	–
PB	<0.020 ± 0.0^a^	5.9 ± 0.16^a^	1.1 ± 0.12^d^	–	0.01 ± 0.17^e^	–
BF	<0.020 ± 0.0^a^	4.2 ± 0.12^c^	0.36 ± 0.11^e^	–	0.56 ± 0.19^b^	–
PPFP	<0.020 ± 0.0^a^	4.2 ± 0.53^c^	3.01 ± 0.13^a^	0.13 ± 0.01	0.26 ± 0.07^d^	
PPDP	<0.020 ± 0.0^a^	5.6 ± 0.15^b^	2.07 ± 0.47^b^	–	6.69 ± 0.16^a^	–

^*a*^Values are mean ± SD of triplicate analysis.

^*b*^Not detected (–).

^*c*^PPF (Pure pride).

^*d*^PB (diet consisted of a mixture of 50% Pure pride and 50% Betagro).

^*e*^BF (Betagro).

^*f*^PPFP (diet consisted of a mixture of Pure pride supplemented with 100 g fresh pumpkin pulp per day).

^*g*^PPDP (diet consisted of a mixture of Pure pride and 100 g dry pulp pumpkin per day).

^*h*^Mean values with different superscripts letters in a column are significant at the 0.05 level.

### Mineral Content of the House Crickets

Crickets mineral content was also significantly influenced by the diet treatments: the highest amount of sodium (*F*_4,10_ = 32888966.92, *P* < 0.001), calcium (*F*_4,10_ = 11049659.12, *P* < 0.001), phosphorus (*F*_4,10_ = 258370288.97, *P* < 0.001), and potassium (*F*_4,10_ = 317530254.22, *P* <0.001) were observed in fed crickets on PPF, the lowest in those fed on BF. However, crickets fed on BF had the highest amount of iron (*F*_4,10_ = 61424.53, *P* < 0.001) compared to PPF, whereas those fed on PPDP had the highest amount of zinc (*F*_4,10_ = 45128.15, *P* < 0.001), the lowest in PPFP (Table 5).

**Table 5.** Mineral content (mg/100 g dry matter basis) of the house crickets on two commercial diets, PPF (22% protein diet) and BF (16% protein diet) and other formulated diets, PB (19% protein diet), PPFP (18% protein diet), and PPDP (20% protein diet)

Diet treatment	Sodium	Calcium	Iron	Phosphorus	Potassium	Zinc
PPF	471.4 ± 0.12^a^	186.9 ± 0.12^a^	3.3 ± 0.17^e^	1038.9 ± 0.12^a^	1211.1 ± 0.14^a^	15.7 ± 0.16^d^
PB	429.3 ± 0.04^b^	176.6 ± 0.34^c^	4.9 ± 0.21^b^	900.2 ± 0.13^b^	1017.6 ± 0.03^b^	22.2 ± 0.18^a^
BF	332.4 ± 0.16^d^	122.8 ± 0.13^d^	7.4 ± 0.16^a^	816.9 ± 0.06^d^	851.2 ± 0.19^d^	19.9 ± 0.34^c^
PPFP	255.3 ± 0.52^e^	99.6 ± 0.51^e^	3.9 ± 0.12^d^	543.4 ± 0.08^e^	644.4 ± 0.21^e^	13.3 ± 0.12^e^
PPDP	399.2 ± 0.97^c^	184.3 ± 0.72^b^	4.7 ± 0.11^c^	837.9 ± 0.13^c^	995.9 ± 0.18^c^	21.7 ± 0.14^b^

^*a*^Values are mean ± SD of triplicate analysis.

^*b*^PPF (Pure pride or 22% protein diet).

^*c*^PB (diet consisted of a mixture of 50% Pure pride and 50% Betagro or 19% protein diet).

^*d*^BF (Betagro or 16% protein diet).

^*e*^PPFP (diet consisted of Pure pride supplemented with 100 g fresh pumpkin pulp per day or 18% protein diet).

^*f*^PPDP (diet consisted of a mixture of Pure pride and 100 g dry pulp pumpkin powder per day or 20% protein diet).

^*g*^Mean values with different superscripts letters in a column are significant at the 0.05 level.

## Discussion

The correlation between cricket diets and improved nutritional value needs to be consolidated with scientific evidence. The results of this present study have been discussed to highlight the effects of two major commercial diets and other formulated diets as well as the effect of either fresh pumpkin pulp or dry pulp pumpkin powder inclusion in a commercial cricket diet on the nutritional composition and growth parameters of *A. domesticus*.

Reporting an appropriate growth pattern of the house crickets is crucial for the development of suitable diets and adaption of diet treatments to reduce cost and maximize yield output. The present study demonstrated the pattern of growth of the house crickets under the five diet treatments: from day 1 to 11, all nymphs, being in the lag phase of growth, exhibited the same pattern of growth (Figs. 1 and 2). However, there were variations in the number of days the nymphs required to become adults; fed crickets on PPDP, PPFP, and PPF had superior growth rates compared to the other groups. This concurs with previous data on this insect species (Patton 1967, Orinda et al. 2017). These studies also indicated that the house crickets thrive very well on 20–30% protein diets and therefore explains the growth performance of the house crickets on these diets. The growth pattern provides scientific evidence for the high growth, and slow growth rate hypothesis from the lag phase to the stationary phase. However, cricket’s width measurements (Fig. 3) cannot be used as a tool to estimate the growth rate, due to large variations observed in this present study. Understanding the influence of diet on cricket’s body size, particularly, body weight, is also important to determine the suitability of the commercial diet for improved income: our results from the feeding experiment revealed a substantial growth compatibility under the diet treatments: crickets reared on PPF (22% protein) had 9% more mean body weight and length compared to BF (16% protein), as normally observed in insects in response to high- and low-protein diets (Orinda et al. 2017). Further, addition of either 100 g fresh pumpkin pulp or 100 g dry pulp pumpkin powder to one of the commercial diets PPF (22% protein) increases the mean body weight and length (Figs. 1 and 2). The pumpkin inclusion and diet may have supported the growth requirement of *A. domesticus* (McFarlane et al. 1959, Cohen 2015). Alternatively, because of the low protein and high carbohydrate contents of the pumpkin (Adebayo et al. 2013, Fagbemi 2019), as reported in previous studies: diet high in carbohydrate and low in protein has been associated with increased weight gain of *A. domesticus* (Orinda et al. 2017). Furthermore, the findings of Floater (1997) had revealed positive impact of water on the growth of insects. Increased access to water can improve the formation of cellular membrane and homeostasis resulting in well-hydrated *A. domesticus* (Alpert 2006). This may have also contributed to the slight variation in the mean body weight of fed crickets on PPF (22% protein) supplemented with either 100 g fresh pumpkin pulp or 100 g dry pulp pumpkin powder compared to those on PPF alone (22% protein).

Crickets surviving rate was equally matched on the five diet treatments and therefore suggested that diet has no effect on *A. domesticus* survival rate. In general, insects have high survival rate (Veenenbos and Oonincx 2017). The main reason crickets in this present study had high survival rate may be due to water provision, which plays a critical role in improving survival rate and physiological functions of crickets (Floater 1997, McCluney and Date 2008), or because crickets were provided with enough diets and reared under good agricultural practice (GAP), this could have a positive impact on survival rate.

Comparing the effect of the different diets on FCR revealed that increasing the protein content of diets enhanced feed efficiency estimated from FCR (Table 2). Those under fed BF (16% protein) had the highest FCR value (1.81 ± 0.02) among the group, indicating that this group is less efficient in converting feed to body size. The inclusion of either fresh pumpkin pulp or dry pulp pumpkin powder to PPF (22% protein) has no effect on FCR, when comparing the results to those on PPF alone. Veenenbos and Oonincx (2017) also found that fed crickets on a suitable protein diet supplemented with carrots resulted in similar FCR values compared to the control. Therefore, the high protein content of the diet (PPF) in this present study (McFarlane et al. 1959), mineral and vitamin contents (Lall et al. 2002) may have contributed to the improved FCR values when compared to those on BF (16% protein), via improving the weight of crickets, since FCR depends on weight gain. However, further research is required to clarify the effects of vitamins and minerals supplementation on growth parameters such as weight and FCR of the house crickets. Additionally, the high crude protein content and amino acid digestibility of PPF (22% protein diet) also explain the low FCR value obtained from fed crickets on PPF (22% protein) compared to BF (16% protein). Further evidence of the positive impact of protein on crickets FCR came from, the Pearson’s correlation analysis, which showed that as crude protein content of the diet increases the FCR values gets smaller (*r* = −0.618, *P* = 0.266). Moreover, the results of the Pearson’s correlation analysis showed that as crude protein content of the diet increases the FCR values gets smaller (*r* = −0.618, *P* = 0.266). 

Cost of feeding per kg live weight gain on a low-protein diet (BF) was significantly lower compared to the other groups, except fed crickets on PB (Table 2). However, the group recorded the lowest mean body weight and the highest quantity of feed consumed. This has huge implication for income, while diet with a high crude protein content may be expensive to produce a kilogram of crickets less amount of diet is required and may result in higher yield output. This may be cheaper compared to diet with a low crude protein content. Crickets have been reported to be high feed convertors (Nakagaki and Defoliart 1991, Finke 2002, Wilkinson 2011), which concurred to the findings in this current study. In general, insects are efficient feed convertors compared to conventional animals; being cold blooded, they can use less energy to maintain homeostasis and prevent the wastage of protein for energy instead of growth (Collavo et al. 2005).

Previous studies have consistently reported that the proximate composition of the crickets is influenced by the diet, especially protein, carbohydrate and fat content of the diet (Finke 2002, Oonincx et al. 2019). We had suspected that crickets will have a high protein and low fat content when fed on a high-protein diet. Not surprisingly, from the five diets evaluated, crickets had high protein and low fat content on a high-protein diet (22% protein). Consequently, the results of previous study that high protein content of diet resulted in high protein and low fat content in crickets are justified (Oonincx et al. 2015). The results also indicated that fat store can be reduced by improving the protein quality of the diet (protein quality of diet is a function of the amount and digestibility). These findings by extension provide the answer to solve global malnutrition, particularly obesity and diet-related diseases. Since high fat store in a fed cricket on low-protein and high-carbohydrate diet may be applicable to human. Therefore, provided a trend to utilize fed crickets on either PPF (22% protein) or PPDP (20% protein) in the promotion and management of type 2 diabetes, obesity, and cardiovascular diseases, due to high protein and low carbohydrate content. Because diet high in protein and low in carbohydrate has been associated with improvement in chronic diet-related diseases (Iqbal et al. 2009, Black et al. 2013, Dyson 2015). Further, the amount of protein in the house crickets in this present study was in accordance to values reported in previous studies (Oonincx et al. 2015, Ayieko et al. 2016), their consumption can improve population dietary protein intake. But dietary protein intake can easily be achieved through the promotion of fed crickets on a 22% protein diet alone or 22% protein and dry pulp pumpkin powder inclusion: 100 g of the house crickets can supply 63 g protein compared to 25.6 g protein in 100 g of beef and 39 g protein in 100 g of chicken (Finke 2002).

Despite the high cost of adding pumpkin to a commercial diet, it was observed that this feeding method was not suitable for improving the protein and fat content in crickets. In addition to low protein content, we observed that the fat content was 80% more than fed crickets on PPF alone (Table 3). The decline in protein content relative to greater percentage of fat may be due to the low protein and a high carbohydrate content of the fresh pumpkin pulp. Because high carbohydrate content of this diet supplied the calories required and promoted the synthesis of fat (Iqbal et al. 2009, Balogun and Olatidoye 2012). In other words, if the protein, carbohydrate, and fat ratio of the insect diet are not well matched to its physiological and physical functions. The excess carbohydrate not utilized for energy will also be stored as fat. This explains the high fat content in fed crickets on a high-protein diet (22% protein) supplemented with fresh pumpkin pulp in this present study. This was probably due to the high carbohydrate and low protein content of the fresh pumpkin pulp (Adebayo et al. 2013, Fagbemi 2019).

Another relevant evidence covered in this present study relates to providing evidence of the influence of the diet treatments on vitamin and mineral content in crickets. We found that the inclusion of fresh pumpkin pulp in commercial diets can be utilized to improve the vitamin B content in crickets, except vitamin B1, due to the low amount, below detection limit, which was observed in this present study. This explains the high amount of vitamin B in this group compared to values reported in previous study (Ayieko et al. 2016). However, vitamin A analysis showed that vitamin A was not detected in *A. domesticus*, indicating that *A. domesticus* are not good sources of vitamin A. These findings were not in agreement with previous published data (Ayieko et al. 2016). The vitamin A content of the house crickets reported in this study was probably, the results from diet in the insect’s gut, containing small quantity of this dietary factor. In this current study, crickets were starved for 8 h and were denied access to water for 4 h before harvesting.

Phosphorus, potassium, calcium, and sodium are the most abundant mineral elements reported in most studies on the nutritional profile of the house crickets (Oonincx et al. 2015, Ayieko et al. 2016), which concurred with the values reported in this study. The trend was more intense for fed crickets on PPF (22% protein). The data obtained from Pearson’s correlation analysis also suggested that high protein content in crickets resulted in high sodium (*r* = 0.866, *P* = 0.001), potassium (*r* = 0.874, *P* = 0.001), phosphorus (*r* = 0.913, *P* = 0.001), and low fat (*r* = −0.987, *P* = 0.001) content. Therefore, the high protein content in *A. domesticus* relative to several mineral elements analyzed in this present study is connected to the significant high sodium, calcium, phosphorus, and potassium in fed *A. domesticus* on PPF. However, the supplementation of high-protein diet (22% protein) with fresh pumpkin pulp (thus, those under fed PPFP) was associated with low protein and high fat content in *A. domesticus* through the ingestion of pumpkin, low-protein and high-carbohydrate diet, over the high-protein diet (PPF). This result indicated that consuming fed crickets on a diet supplemented with fresh pumpkin will allay protein and mineral benefits. Oonincx et al. (2015) also found low protein content in house crickets fed on a suitable protein diet supplemented with carrots. However, dry pulp pumpkin powder inclusion resulted in improved protein content compared to fresh pumpkin pulp. The increase in protein content via the inclusion of dry pulp pumpkin powder might be due to increase in protein content of the pumpkin after drying (Fagbemi 2019). Fed crickets on PPDP were not provided with the option to either the commercial diet or the dry pulp pumpkin powder as this diet was thoroughly mixed. Therefore, the continual ingestion of the dry pulp pumpkin powder over the high-protein diet (PPF), is not possible, as with the supplementation of the fresh pumpkin pulp.

When comparing mineral elements in all treatments, it was realized that iron was the lowest mineral element in the house crickets. This trend was very noticeable in fed crickets on PPF (22% protein). However, there is clear evidence that the RDI can also be achieved through the promotion of fed crickets on this diet (22% protein) compared to the consumption of widely consumed animals such as beef and chicken. The usage is based on the fact that the mean iron content of fed crickets on this diet treatment is 27% more than the iron content of 2.4 mg in 100 g of beef and 64% more than the iron value of 1.2 mg in 100 g of chicken (Finke 2002). Possible differences in the ingredient used in the diet’s formulation, especially ground used as ingredient in BF. The usage might have resulted in high iron content in the diet and could explain the difference in the iron content. Therefore, broadens our understanding of the hypothesis that high protein contents of diet may lead to high protein content in the house crickets is tenable for other nutrients: fat, vitamins, and minerals in crickets may also be influenced by their relative amount in the diet. In general, protein and mineral content of house crickets are high when fed on a high protein diet (Rumpold and Schlüter 2013, Oonincx et al. 2015). This concurs with the findings of this present study. Further, Oonincx et al. (2019) found a greater amount of omega-3 fatty acid in fed crickets on a diet supplemented with this dietary factor (omega-3 fatty) compared to the control. This provided a trend to usefully improve the nutritional profile of the house crickets via the formulation of suitable diets.

In conclusion, the results suggested that supplementation of 22% protein diet with either fresh pumpkin pulp or dry pulp pumpkin powder enhanced feed utilization estimated from FCR and improved weight gain (bigger size crickets). Fed crickets on a 22% protein had the highest amount of protein and several minerals, such as phosphorus, potassium, calcium, and sodium. Although the cost of dry pulp pumpkin powder inclusion to 22% protein diet was the highest among the diet treatments evaluated, it is worth mention that crickets on this treatment had the highest amount of vitamin B3 and B12. However, rearing crickets on a 22% protein diet supplemented with fresh pumpkin pulp will lead to low protein, high fat, and low mineral, such as sodium, calcium, phosphorus, and potassium. We found that diet has no significant effect on crickets surviving rate. Crickets can efficiently be reared on a 22% protein diet to maximize profit and improve mineral contents in crickets, whereas as the supplementation of 22% protein diet with dry pulp pumpkin powder will improve vitamin B content in crickets.
